# Aquaporins: New players in breast cancer progression and treatment response

**DOI:** 10.3389/fonc.2022.988119

**Published:** 2022-09-21

**Authors:** Verodia Charlestin, Daniel Fulkerson, Carlos E. Arias Matus, Zachary T. Walker, Kevin Carthy, Laurie E. Littlepage

**Affiliations:** ^1^Department of Chemistry and Biochemistry, University of Notre Dame, Notre Dame, IN, United States; ^2^Harper Cancer Research Institute, University of Notre Dame, South Bend, IN, United States; ^3^Department of Biotechnology, Universidad Popular Autónoma del Estado de Puebla, Pue, Mexico

**Keywords:** aquaporin, breast cancer, metabolism, structure, channel, glycerol

## Abstract

Aquaporins (AQPs) are a family of small transmembrane proteins that selectively transport water and other small molecules and ions following an osmotic gradient across cell plasma membranes. This enables them to regulate numerous functions including water homeostasis, fat metabolism, proliferation, migration, and adhesion. Previous structural and functional studies highlight a strong biological relationship between AQP protein expression, localization, and key biological functions in normal and cancer tissues, where aberrant AQP expression correlates with tumorigenesis and metastasis. In this review, we discuss the roles of AQP1, AQP3, AQP4, AQP5, and AQP7 in breast cancer progression and metastasis, including the role of AQPs in the tumor microenvironment, to highlight potential contributions of stromal-derived to epithelial-derived AQPs to breast cancer. Emerging evidence identifies AQPs as predictors of response to cancer therapy and as targets for increasing their sensitivity to treatment. However, these studies have not evaluated the requirements for protein structure on AQP function within the context of breast cancer. We also examine how AQPs contribute to a patient’s response to cancer treatment, existing AQP inhibitors and how AQPs could serve as novel predictive biomarkers of therapy response in breast cancer. Future studies also should evaluate AQP redundancy and compensation as mechanisms used to overcome aberrant AQP function. This review highlights the need for additional research into how AQPs contribute molecularly to therapeutic resistance and by altering the tumor microenvironment.

## Introduction

Aquaporins (AQPs) are a family of small transmembrane proteins that facilitate the transport of water across plasma cell membranes (1–3). AQPs can also transport glycerol, urea, gases, and other small molecules, which regulate numerous cellular functions, including energy metabolism, migration, immunity, barrier function, angiogenesis, and osmotic water movement (2, 4).

Currently, the family includes at least 13 mammalian AQP family members in higher mammals that are classified based on structural and functional characteristics and can be divided primarily into two subfamilies: AQPs that are *water selective* (AQPs 0-2, 4-6, and 8) and aquaglyceroporins that are *glycerol permeable* (AQPs 3, 7, 9, and 10) (1, 3). Control of glycerol transport by aquaglyceroporins plays a crucial role with the dysregulation of these glycerol channels being associated with metabolic diseases, such as obesity, insulin resistance, and cancer (5, 6). In addition to these subfamilies, AQP11 and AQP12, the most distantly related paralogs, have low sequence similarity with other AQPs and localize to the membrane of intracellular organelles instead of the plasma membrane (7, 8). Due to their subcellular localization and sequence features, AQP11 and AQP12 are classified as *S-aquaporins* (7, 8).

Studies that began evaluating multiple AQP family members in cell-based assays and multiple cell types have contributed significantly to our understanding of the functional complexity and significant differences between these water channel proteins. Structural and functional studies have been critically important in highlighting how AQP regulation, such as through post-translational modifications like phosphorylation, differentially regulates mammalian AQPs and alters AQP function, including gating, trafficking, and protein-protein interactions (9). Such studies highlight the need to further understand how AQPs can be regulated with post-translational modifications and differential regulation of individual AQPs within the same cell. The noted differences in AQP structure, functional properties, mechanisms of regulation, and tissue-specific distributions highlight the importance of understanding each AQP within defined physiological contexts, both in normal developmental processes and in human disorders, especially since each AQP and each cellular context may vary. Protein-protein interactions also critically contribute to AQP regulation and mediating alternative functions (10). Together, these studies have significantly advanced our understanding of the diverse roles that AQPs play in health and disease and how their regulation, particularly through post-translational modifications and signaling mechanisms, control AQP activation, gating, trafficking, and participation of AQPs in signal transduction pathways (4, 9, 11).

Over the last decade, a growing interest in AQPs has identified AQPs as targets for drug discovery, as players in cancer biology, and as diagnostic and therapeutic targets in cancer (12–16). Increasing evidence links AQP expression with key biological functions in cancer, where aberrant AQP expression correlates with altered proliferation, tumor type, grade, and prognosis (14, 15, 17). In breast cancer, AQP expression is significantly associated with overall survival and relapse free survival (18, 19). AQPs impact tumor growth and metastasis by regulating proliferation, migration, and angiogenesis in mammary tumors and breast cancer cell lines (12, 19–21). Although we are beginning to understand the importance of AQPs in cancer, further elucidation of AQP structure and function will shed light into the additional and unexpected roles of these transport proteins and how their dysregulation can lead to disease. Here, we review the role of AQPs in breast tissue and breast cancer, the tumor microenvironment, and their impact and future implications on cancer progression, metastasis, and treatment as predictive biomarkers and therapeutic targets.

## Aquaporin structure and function

A number of reviews and articles provide detailed information on the structural and functional characterization of AQPs (1, 3, 22–24). We include a generalized overview of AQP structure and function and primarily focus on key structural features that may be relevant to breast cancer.

**Figure 1:**
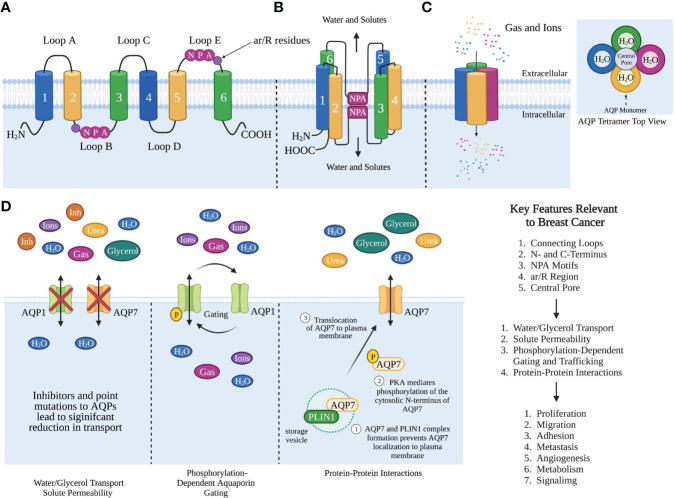
Aquaporin Structure and Function Relevant to Breast Cancer. **(A)** Cartoon representation of AQP monomer topology. The transmembrane helices 1-6, extracellular loops **(A, C, E)**, intracellular loops **(B, D)**. Singled-letter N-P-A codes in pink circles mark the NPA motifs and ar/R in purple circles. **(B)** Organization of helices and NPA motifs on the loops bend to pair with each other and form the water channel monomer. **(C)** AQP monomers assemble as homotetramers to form a central pore. The structure of AQP homotetramer from the top view shows each AQP monomer containing an independent water pore. **(D)** AQP functions that are relevant to breast cancer. Created with BioRender.com. Figures **(B, C)** were modified from Reference (12) with permission. Figure **(D)** was modified from reference (9) with permission

AQPs are tetrameric proteins, where each monomer consists of six alpha-helical domains that span the membrane and two half membrane-spanning helices that surround a narrow aqueous pore (**Figures 1A, B**) (3, 25). The six alpha-helical domains are connected by five loops, three extracellular loops, loops A, C, and E, while loops B and D are intracellular (25, 26). AQPs also contain intracellular amino and carboxyl termini. The monomers interact with one another to form a tetramer with a central pore (**Figure 1C**).

AQPs primarily function as bi-directional water transporters, where each monomer functions independently as a water channel (27). Aquaglyceroporins are a subset of AQPs that can transport both water and glycerol. Although glycerol can cross the lipid bilayer *via* passive diffusion, the presence of aquaglyceroporins facilitates increased glycerol permeability thus regulating glycerol transport. AQPs mediate *water transport* with different efficiencies, with the aquaglyceroporins being poor water facilitators compared to orthodox aquaporins, with AQP7 showing the lowest water permeation (28). However, the *glycerol transport* efficiency among the AQPs is highest for AQP7, with AQP3 and AQP9 transporting half the amount of glycerol compared to AQP7.

In addition to water and glycerol transport, AQPs also transport small molecules, gases, hydrogen peroxide, ammonia, urea, and arsenite (**Figure 1D**) (29–32). Cells expressing AQPs on the plasma membrane have a 5-50 fold higher osmotic permeability and faster AQP-dependent gas transport than free diffusion, implicating a key role for AQPs in maintaining biological function and transport of these biological molecules (1, 33). Since AQPs transport not only water but also small molecules and solutes, they regulate a number of functions, such as water homeostasis, exocrine gland secretion, urine concentration, skin moisturization, fat metabolism to cell proliferation, migration, and adhesion (33–35).

Water/glycerol transport can be regulated by hormones, phosphorylation, intercellular pH, and calcium levels, which have been summarized in several recent reviews (4, 9, 36). We will discuss relevant aspects of regulation to AQP function and structure. Uptake studies in yeast strains overexpressing human AQP3, AQP7, and AQP9 showed accumulated ^14^C-glycerol at a pH of 7.5 (28). AQP7 was the most prominent glycerol transporter compared to AQP9 and AQP3. Changes in pH from 7.5 to 6 significantly decreased the glycerol transport of AQP3 but did not affect AQP7 or AQP9 (28). With lowered pH (~5.5), AQP3, AQP7, and AQP9 did not allow glycerol permeation, while hAQP10 did allow glycerol flux in a fluorescent based assay. However, at pH 7.4, AQP10 did not allow glycerol permeation (37). This result was inconsistent with another study that observed that hAQP10 allows glycerol permeation at neutral pH using a scintillation counter to measure ^14^C-glycerol uptake (38). Since cancer cells have an altered pH compared to normal cells, the changes in pH may also alter AQP function and should evaluated.

### Mechanisms of permeation and mutational analysis of AQPs

Although AQPs share the same structural architecture, any variations in key structural features can impact their functional characteristics like selectivity and gating. AQP structure-function analysis has provided insight into the importance of these structural features and their functional implications. We review the conserved structural features that are critical for AQP function: the intra- and extracellular loops, the asparagine-proline-alanine (NPA) motifs, the aromatic-arginine (ar/R) regions, and the central pore.

#### Connecting loops

The intracellular D loop/loop D stabilizes the tetramer and acts from the cytoplasm as a gate to the central pore. In the closed form, the D loop blocks the central pore, and in the open form, the D loop does not. Molecular dynamics studies in plant AQPs show that the N terminus interacts with the D loop through hydrogen bonding (39). Upon phosphorylation, the bond dissociates and the loop undergoes a conformational change that opens the pore (39, 40).

The D loop also is predicted to contribute to water permeability and to regulate AQP oligomerization (41, 42). Recent work has highlighted the importance of the tetrameric structure in plasma membrane localization and transport function. Interestingly, tetrameric assembly of AQP4 is not required for either endoplasmic reticulum and Golgi trafficking to the plasma membrane or single channel water activity (41). However, mutations in the D loop of AQP4 that cause reduced tetrameric assembly were unable to localize to the plasma membrane in response to a hypotonic extracellular stimulus, indicating a need for tetrameric assembly. Similar mutations in the D loop of AQP1 caused similar changes in oligomerization. AQP1 and AQP3 exist primarily as a tetramer and monomer, respectively, while D loop loss and gain mutations caused migration as a monomer and dimer, respectively (41). Additional mutagenesis of AQP1 in loops B and E demonstrated that residues in these regions are critical for tetramer assembly and water permeability, with loop B playing a more critical role than loop E in oligomerization (43). Together, this evidence supports a critical role for the connecting loops in AQP tetramer formation, which affects both trafficking of AQPs to the plasma membrane and water permeability (41).

#### NPA motifs

Each AQP monomer contains two highly conserved NPA sequence motifs that lie in the middle of the channel, located in loops B and E, to form a central constriction (**Figures 1A, B**) (27, 44). The NPA sequence motif is critical for water and substrate permeation (27).

Several AQPs, including AQP7, AQP11, and AQP12, contain variations in the NPA sequence motif. In AQP7, the first NPA motif is substituted by an NAA (asparagine–alanine–alanine) motif, and the second NPA motif is substituted by an NPS (asparagine–proline–serine) (45, 46). In contrast, AQP11 and AQP12 have only one amino acid difference in the first NPA sequence: NPC (asparagine-proline-cysteine) in AQP11 and NPT (asparagine-proline-threonine) in AQP12 (47).

Since all AQPs conserve the asparagine residue, this may emphasize its importance for function. Mutagenesis studies on these conserved NPA sequence motifs indicate their importance.

In AQP1, NPA deletions did not affect the expression and intracellular processing of AQP1, given that the membrane expression pattern of all NPA deletion motifs were similar to wildtype (WT) AQP1 (48). However, functional analysis showed that deleting NPA motif 1 and NPA motif 2 reduced water permeability by 49.6% and 46.7%, respectively, while NPA double deletion had little effect on AQP1 water permeability. This suggests that NPA motifs are important for water permeation but not essential for the expression and intracellular processing of AQP1. Another study showed that mutations within or adjacent to NPA motifs reduced water permeability and localization to the plasma membrane (27). The most drastic changes were observed in aspartic acid and glutamine substitutions of asparagine (N76Q, N192D, and N192Q) in AQP1 where mutants had significantly reduced water permeability. A significant reduction in plasma membrane localization was also noted for N192Q, N192Q/D237Z, and A194V, indicating a critical role for the conserved sequences in the NPA motif (27).

Similarly, deletion of one or both NPA motifs in AQP4 also decreased plasma membrane trafficking and increased retention in the endoplasmic reticulum (49). However, neither mutation influenced AQP4 protein synthesis or degradation.

In AQP11, point mutation to the NPC motif, C101A, did not impact the subcellular localization of AQP11 (50). However, the C101A mutation reduced the oligomerization of AQP11 and was required for AQP11 water permeability.

#### ar/R regions

Located on the extracellular side, the aromatic/arginine (ar/R) region is formed by conserved arginine and aromatic residues, like phenylalanine and tryptophan. Narrower than the central NPA constriction, the ar/R region provides pore selectivity by size exclusion and positive charge repulsion by the arginine. The ar/R region is a conserved difference between aquaporins and aquaglyceroporins, where the ar/R constriction is wider and less polar in aquaglyceroporins than in aquaporins to allow glycerol and urea permeability.

In AQP7, the ar/R region, which results from F74-R229 interaction, is reinforced by the presence of an extra aromatic residue Y223, thereby forming an ar^2^/R region with stronger cation-π interaction (38). In AQP7, point mutations to the ar^2^/R region (Y223I, Y223A, R229A, and F74G/Y233A), extracellular vestibule (N220A and N220D), and intracellular gate (D191A, Q192A, E193A) significantly reduced glycerol permeability (38). Although the R229A point mutation in AQP7 did not impact AQP7 stability or tetramer assembly, it significantly weakened the binding of glycerol to AQP7, indicating the ar^2^/R serves as both a substrate binding site and a gate for glycerol transportation.

AQP10 is the only aquaporin with no aromatic residues in the ar/R gating domain, while all the others will have phenylalanine, tyrosine, or tryptophan in one or two positions of the ar/R domain (38, 51).

Double point mutations to the ar/R of rat AQP1 (F56A/H180A) enabled both glycerol and urea passage by enlarging the diameter of the ar/R constriction three-fold (52), whereas H180A/R195V double point mutant enabled only urea passage. Single- and double-point mutations to the ar/R of AQP1 (H180A, R195V, F56A/H180A, and H180A/R195V) allowed ammonia permeability. Removal of the positive charge in the ar/R constriction of AQP1 (R195V and H180A/R195V) allowed the passage of protons through AQP1 (52).

#### Central pore

Even though AQPs have been shown to permeate gas and ions, the pathway for transport through the central pore or monomer remains elusive. Early reports suggest that this pore transports ions and gases in some AQPs. Recent observations have found that carbon dioxide (CO_2_) moves through the monomeric and central pores of AQP1, although the central pore has a greater CO_2_ permeability than all four monomers combined (36, 53). The central pore of AQP1 is also permeable to nitric oxide (NO), Na^+^, K^+^, and Cs^+^ (4). The gating may be regulated by cGMP and phosphorylation (36). With AQP1, cGMP binding causes a conformational change that pushes the D loop out of the pore (54). Mutational studies of arginine residues (R159A and R160A) in loop D had no effect on water permeability; however, ion channel conductance was significantly reduced in response to increased cGMP (54).

Like AQP1, AQP4 conducts gas molecules NO and O_2_, where the central pore is more conductive than the monomer (55). Although longer and narrower than AQP1, AQP4 more readily conducts gas molecules compared to AQP1 in the brain using various simulation techniques. This is because it provides a more energetically favorable permeation for gas molecules due to the orientation of charged residues near the pore entrance. AQP4 also acts as an NO reservoir (55).

By molecular dynamics, CO_2_ can permeate AQP5 with higher permeation of CO_2_ through the central pore than the four monomers (56). Unlike other AQPs, AQP5 contains a lipid residue, phosphatidylserine (PS6), in its central pore (57, 58). Molecular dynamics studies on the effects of PS6 showed that the central pore does not alter structure or water transport function (57). However, PS6 inhibited gas permeation by occluding the central pore, preventing gas permeation.

Studies have yet to determine whether these structural features and regulatory mechanisms have a role in breast cancer progression and metastasis. Given the importance of these structural features and their functional implications this would suggest a potential mechanism in which AQPs impact breast cancer (**Figure 1**). AQPs could contribute to proliferation and metastasis through their transport function or as an alternative mechanism to transport metabolites.

## Aquaporin expression and localization in normal adipose tissue and mammary gland

AQPs are widely distributed throughout the body and are expressed in epithelial, endothelial, and other cell types (34). Since the mature breast is composed of bilayered epithelial cells and is surrounded by the extracellular stroma that includes large amounts of adipose tissue/adipocytes, we focus on AQP expression and localization in normal tissue in adipose tissue as well as in the mammary gland.

### Adipocytes

Adipocytes play a role in maintaining energy balance. They synthesize and hydrolyze triacylglycerols (TAGs) and are a key source of plasma glycerol. Adipose tissue can be classified by its location and functional characteristics. In terms of location, subcutaneous adipose tissue lies beneath the skin, whereas visceral adipose tissue lines internal organs. In terms of physiology and function, adipose tissue is divided into three classes: white adipose tissue (WAT), beige adipose tissue (BeAT), and brown adipose tissue (BAT) (59, 60). WAT is key for energy storage, endocrine communication, and insulin sensitivity, whereas BAT is involved in body thermogenesis. WAT is distributed throughout the body in subcutaneous regions and surrounds visceral organs. BeAT shares some similar properties and marker expression to BAT but is developmentally derived from different precursor cells and activated by different signals (60). BeATs are located within subcutaneous WAT and are developmentally derived from WAT.

Adipocytes play a key role in lipid and glucose metabolism, where adipocytes synthesize TAGs from free fatty acids (FFA) and glycerol-3-phosphate in a process called lipogenesis (61). During lipolysis, TAGs are hydrolyzed to glycerol and FFA in adipocytes. Energy balance and glycerol transport by AQPs in these cells are maintained by lipogenesis and lipolysis. AQP7 is a well-studied gatekeeper of glycerol transport in adipose and other tissues (62–64). *Aqp7* deficiency in mouse models is associated with adipocyte hypertrophy, increased glycerol and triglyceride accumulation, insulin resistance, and increased obesity in both mice and humans (65, 66). During high energy demands and metabolic stress, lipolysis increases and converts triglycerides into free fatty acids and glycerol. AQP7 controls the efflux of glycerol under these conditions. AQP7 expression increases during lipolysis, including fasting and insulin deficiency, which keeps the rates of glycerol efflux high. When exposed to insulin, AQP7 expression decreases and glycerol transport is reduced (67, 68). Exported glycerol then is taken up by other cells and used as a backbone for energy needs during high energy demands.

AQPs remain the major transporter of glycerol in adipocytes. In fact, the main functions of AQPs in adipocytes are to control glycerol uptake and release, which are essential for lipogenesis and lipolysis (69). The AQP family members are differentially expressed in adipocytes and related cellular structures of adipose tissue (59). AQP7 is the most common aquaglyceroporin in adipose tissue and predominantly is expressed in the plasma membrane and capillary endothelium of adipose tissue (70, 71). We also evaluated Aqp7 expression in normal mouse mammary glands by immunohistochemistry and found Aqp7 protein expressed in both adipocytes and mammary gland epithelium (19). Other AQPs also are expressed in adipose tissue. AQP3, AQP9, AQP10, and AQP11 are also expressed in human adipocytes and function as additional channels of glycerol efflux (61). AQP10 is also present in both human adipocytes and subcutaneous adipose tissue by RT-qPCR and immunoblotting (72). In beige adipocytes, AQP5 and AQP7 were the most highly expressed AQPs by RT-qPCR, followed by AQP9 and AQP3 (59). In white adipocytes, AQP7 was the most expressed AQP, followed by AQP5, AQP9, and AQP3. AQP3, AQP7, and AQP9 were also expressed in human omental and particularly in the subcutaneous adipose tissue by western blot analysis and immunohistochemical expression (73). Although mRNA expression of all three AQPs was similar in subcutaneous tissue, AQP3 was the most abundantly expressed in the omentum followed by AQP9 and then AQP7 (73). In another study, gene expression analysis by RT-qPCR confirmed AQP3, AQP7, AQP9, and AQP10 expression in human abdominal subcutaneous adipose tissue (74). Interestingly AQP1 was significantly expressed, while AQP2, AQP4, AQP5, AQP6, AQP8, AQP11, and AQP12 were not expressed at all.

AQP3 localized to the plasma membrane and cytoplasm of 3T3-L1 cells, whereas AQP7 was predominately in the cytoplasm and AQP9 was constitutively expressed at the plasma membrane in 3T3-L1 adipocytes (73). Immunostaining of 3T3-L1 cells after insulin treatment increased AQP3 detection around lipid droplets, whereas AQP9 was detected in the plasma membrane. Isoproterenol stimulation resulted in AQP3 detection on the plasma membrane. The cellular localization of AQPs also changes in response to also changes in response to extracellular signaling events. For example, AQP7 cellular localization is regulated by protein kinase A (PKA) and comparative gene identification 58 (CGI-58) (70, 75). PKA controls AQP7 translocation and glycerol release by regulating its interaction with Perilipin 1 (PLIN1), a regulator of lipolysis and triacylglyceride mobilization (75). During fasting, PKA phosphorylates the N-terminus of AQP7, which reduces the complex formation of AQP7 and PLIN1 and allows translocation of AQP7 to the plasma membrane for efficient efflux of glycerol. CGI-58, a binding partner of PLIN1 and facilitator of lipolysis on lipid droplets by interacting with adipose triglyceride lipase (ATGL), regulates the translocation and internalization of AQP7 from cortical to intracellular membranes during lipolysis (70). When CGI-58 overexpression decreases ATGL activity and induces AQP7 internalization (70). Cellular localization of AQPs also contributes to AQP function. During lipolysis, AQP localization on the plasma membrane increases to allow glycerol efflux, while in lipogenesis, AQPs traffic to intracellular compartments or around lipid droplets, thus preventing glycerol flux (6, 72, 73, 75).

Much like AQP7, AQP10 localizes to the plasma membrane and cytoplasm and plays a similar role in water and glycerol efflux. AQP10 serves as an alternative pathway for glycerol efflux and, with AQP3 and AQP7, ensures glycerol export from adipocytes (72). Like AQP7, AQP10 cellular localization is regulated by insulin (lipogenic) and isoproterenol (lipolytic) to control fat accumulation. Immunofluorescence of human adipocytes after insulin treatment increased AQP10 staining around lipid droplets, where isoproterenol treatment increased plasma membrane staining but decreased lipid droplet labeling. Although the exact mechanism for trafficking is still unknown, membrane trafficking of AQP10 is similar to AQP7 (72). Interestingly, insulin treatment of human adipocytes did not affect glycerol export. However, isoproterenol-treated adipocytes stimulated glycerol release (37).

Gene expression analysis indicated that AQP11 expression was identified from both subcutaneous and visceral human mature adipocytes (76). AQP11 was identified on the surface of lipid droplets and colocalized with perilipin in the vicinity of lipid droplets by immunofluorescence (76).

### Mammary epithelial cells

AQP protein expression in mammary glands was examined by immunohistochemistry, where AQP1 and AQP3 were expressed in rat, mouse, bovine, and human mammary glands (77–79). RT-qPCR of lactating rat mammary gland also detected AQP4, AQP5, AQP7, and AQP9 gene expression but did not detect AQP2, AQP6, and AQP8 (79). AQP1 localized to both the apical and basolateral membranes of capillary endothelia in the rodent mammary gland by immunohistochemistry. AQP3 localized to the basolateral membranes of secretory epithelial cells and intralobular and interlobular duct epithelial cells immunohistochemically in rat and mouse mammary tissue (79). AQP1, AQP3, AQP4, AQP5, and AQP7 were identified in bovine mammary glands by immunohistochemistry (78). AQP2 and AQP6 were not detected, and AQP9 was only seen in leukocytes within the mammary gland.

We also investigated Aqp7 transcription, protein expression, and localization by immunohistochemistry in normal mammary tissues (19). Aqp7 protein was prominently expressed in both normal adult mouse mammary gland epithelium and adipocytes. We also evaluated Aqp7 expression during developmental stages and found that during lactation, Aqp7 was particularly localized to the plasma membrane compared to during other stages of development.

## Aquaporins in breast cancer

AQPs play an important role in cellular functions associated with cancer progression, such as cell proliferation, cell differentiation, cell migration, and cell adhesion. Various studies have investigated the significance of AQPs in breast cancer. The clinicopathologic parameters and prognostic values of AQPs have been investigated across multiple online databases of human cancer tissue. Gene expression patterns themselves have at times been contradictory, suggesting both pro- and anti-tumorigenic roles for AQPs. The mRNA expression levels of AQP8, AQP9, and AQP10 were upregulated, while those of AQP3, AQP4, AQP5, and especially AQP7, were downregulated in breast cancer based on the Oncomine database (18). In contrast, analysis of relapse free survival (RFS) using the Kaplan-Meier Plotter indicated that high mRNA expression levels of AQP0, AQP1, AQP2, AQP4, AQP6, AQP8, AQP10, and AQP11 were associated with better RFS. AQP3 and AQP9 were associated with worse RFS in breast cancer patients (18). The differences in gene expression may mean that the AQP protein expression or localization, rather than gene expression, is more important in evaluating AQP function within breast cancer.

The gene expression of other AQPs has also been compared in normal and breast cancer tissues. RT-qPCR of breast cancer and corresponding normal tissue did not detect the expression of AQP0, AQP2, and AQP6-9 mRNA (80). AQP10-12 mRNAs were present but were similarly expressed in both normal and cancer tissues. AQP1, AQP3, and AQP5 mRNA expression significantly increased in breast cancer tissue compared to normal tissue and localized to the cell membranes by immunohistochemistry (80).

Other studies find differential expression of the individual AQPs in normal and cancer mammary tissue, with more differences highlighted by comparing gene and protein expression. Our study on the role of AQP7 in breast cancer evaluated AQP7 expression in human and mouse breast cancer and normal tissues (19). Patients with high expression of AQP7 had reduced overall survival compared to tumors with low AQP7 expression, contradicting a prior report (18) that suggested that AQP7 gene expression was protective. In mouse samples by RT-qPCR, Aqp7 expression was higher in normal mammary tissue than in mouse tumors. Aqp7 protein localization was heterogeneous and varied across murine mammary cancer tumor types (19). C3-Tag tumor models had the highest expression of Aqp7, while MMTV-Neu and MMTV-PyMT tumors expressed low levels of Aqp7. MMTV-Wnt1 tumors also expressed low levels of Aqp7 but higher levels in more differentiated tumor tissue, where it localized to the epithelium most proximal to the stroma. However, Aqp7 is often clearly localized to the plasma membrane of mammary cancer cells compared to normal mammary tissue across these tumor types (19). The heterogeneous AQP7 expression results may highlight the value of AQP7 protein levels and localization or activity as better prognostic biomarkers than mRNA expression.

Both expression and cellular localization significantly contribute to the role played by AQPs in breast cancer progression, likely by regulating AQP activity or its regulation. Most of the studies have captured snapshots of AQP localization, rather than evaluating AQP localization in response to growth factor signaling, for example. AQP1 localized to the cytoplasm of cancer cells of invasive breast cancer patients, and cytoplasmic expression of AQP1 promoted breast cancer and correlated with tumor size, distant metastasis, and histological grade (81). In MCF-7 and MDA-MB-231 cells, cytoplasmic staining of AQP1 confirmed cytoplasmic localization (81). Stimulation with epidermal growth factor (EGF) induced redistribution of AQP1 from the cytoplasm to the cell membrane, suggesting ligand-dependent changes in localization. In functional studies in both MCF-7 and MDA-MB-231 cells, overexpressing AQP1 increased proliferation, invasion, and colony formation compared to control cells. AQP3 and AQP5 were detected in human breast cancer tissues, specifically in the membrane and cytoplasm of triple negative breast cancer (TNBC) patients, with AQP5 highly expressed in the invasive front of the tumors (82). In contrast, both AQP3 and AQP5 were poorly expressed in adjacent normal tissue in the periductal and intralobular stroma, endothelial cells, and peripheral nerve fibers by immunostaining, suggesting that expression levels and localization can affect their functional status. Immunohistochemistry of AQP4 indicated that AQP4 protein was more highly expressed in normal breast tissues than in cancer, where AQP4 localized to the membrane and cytoplasm (80).

**Figure 2:**
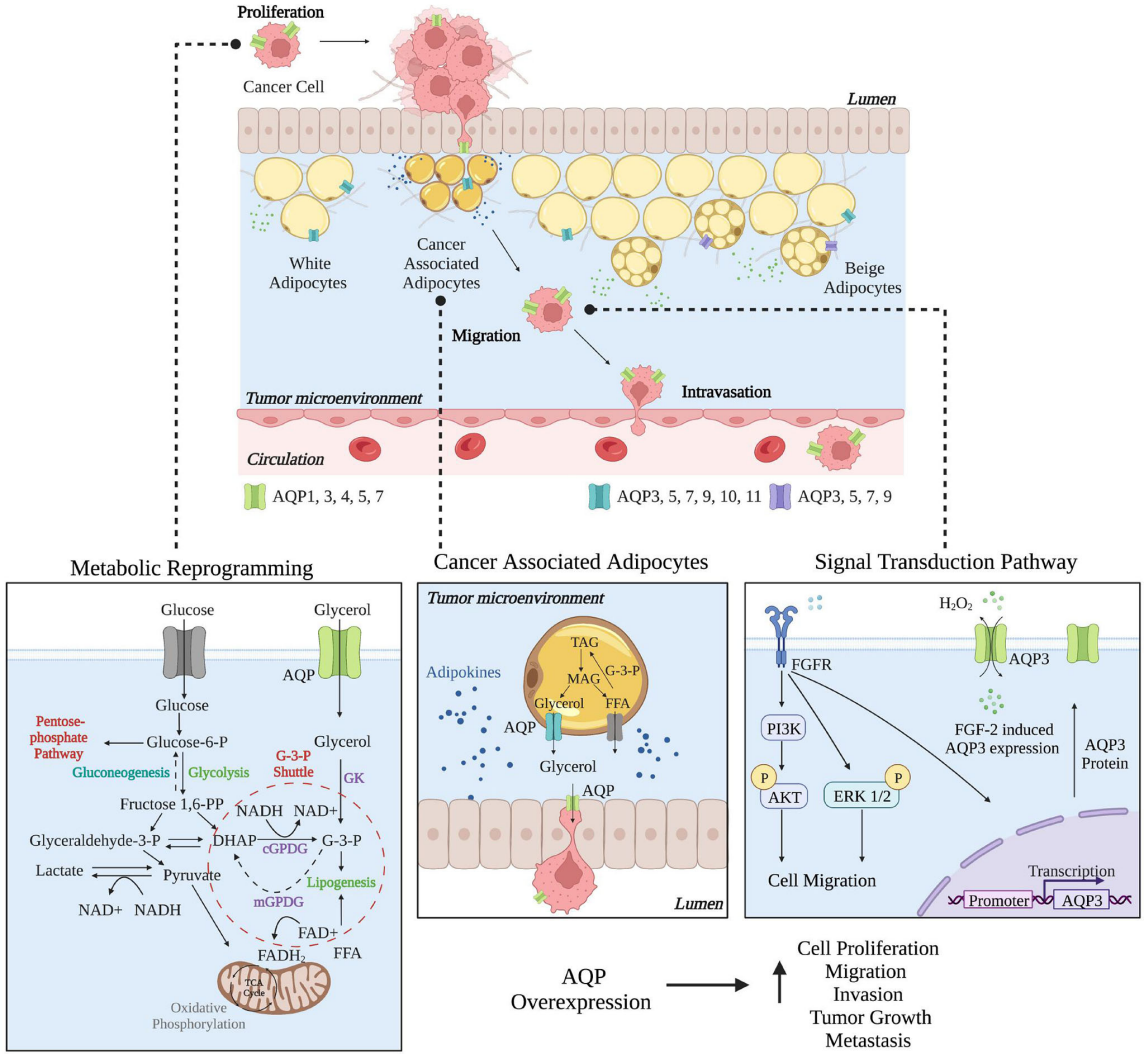
Roles of Aquaporins in Breast Cancer. In breast cancer, individual AQPs have multiple roles, from proliferation to migration to intravasation, where the cancer cells then metastasize to a secondary site. Metabolic reprogramming of cancer cells helps to support proliferation as AQPs can help to facilitate glycerol transport. In cancer-associated adipocytes (CAAs), AQP expression may be essential in facilitating glycerol transport between CAAs and cancer cells. AQP3 expression is critical in cell migration where changes in signal transduction pathways facilitate migration and AQP3 overexpression. Created with BioRender.com. This figure was influenced by Aikman et al. (83).

Next, we outline the roles in breast cancer of AQP1, AQP3, AQP4, AQP5, and AQP7, which are the only AQPs that have begun to be characterized as having roles in breast cancer progression and as potential prognostic markers. **Figure 2** highlights the role AQPs play in facilitating breast cancer from metabolic reprogramming to signal transduction *via* their transport function. **Table 1** provides an overview of the different AQP proteins and their possible related functions in breast cancer.

**Table 1:** Aquaporin Expression in Breast Cancer. AQP expression in breast cancer and their correlated functions.

Correlated Function	References
Not studied in breast cancer but correlated with tumor prognosis	(18, 80)
Cell proliferation, migration, invasion, tumor growth, metastasis, angiogenesis and chemosensitivity	(18, 80, 84–91)
Not studied in breast cancer but correlated with tumor prognosis	(18, 80)
Cell proliferation, migration, invasion, tumor growth, metastasis, angiogenesis, and epithelial-mesenchymal transition	(18, 80, 82, 89, 92–96)
Cell proliferation, migration, invasion, correlated with tumor prognosis	(18, 80, 97)
Cell proliferation, migration, invasion, differentiation, tumor growth, metastasis, angiogenesis, epithelial-mesenchymal transition, and chemosensitivity	(18, 80, 82, 89, 98–103)
Not studied in breast cancer but correlated with tumor prognosis	(18, 80)
Cell proliferation, adhesion, contact inhibition, tumor growth, metastasis, metabolism	(18, 19, 80)
Not studied in breast cancer but correlated with tumor prognosis	(18, 80)
Immune infiltration and correlated with tumor prognosis	(18, 80, 104)
Not studied in breast cancer but correlated with tumor prognosis	(18, 80)
Not studied in breast cancer but correlated with tumor prognosis	(18, 80)
Not studied in breast cancer	(18, 80)

### Aquaporin-1

AQP1 overexpression plays a key role in proliferation, migration, and invasion in several breast cancer subtypes. In TNBC, AQP1 expression was associated with poor survival, where high AQP1 expression was associated with high tumor grade and hormone receptor negativity (84). AQP1 knockdown in MDA-MB-231 inhibited proliferation, migration, invasion, and tumor growth (85). In addition, cytoplasmic expression of AQP1 negatively correlated with prognosis but positively correlated with histological grade, tumor size, lymph node metastasis, and recurrence or distant metastasis (81). AQP1 depletion in MMTV-PyVT mice reduced both tumor growth and lung metastasis (86). Also, AQP1 was a key player in tumor angiogenesis, where AQP1 depletion caused abnormal tumor microvasculature.

Some miRNAs target AQP1 and, thus, reduce breast cancer progression, cell migration, and invasiveness (87, 88). MicroRNAs (miRNAs) are a class of tiny noncoding RNA molecules that regulate gene expression by either directly degrading mRNA or by indirectly repressing protein translation at the post-transcriptional level. In breast cancer patients, miR-320 was significantly reduced in both plasma and tissue samples compared to normal tissue (88). Consistently, patient samples with increased AQP1 expression had reduced miR-320 expression, suggesting a negative correlation between AQP1 and miR-320 expression. Overexpression of miR-320 inhibited cell proliferation, migration, and invasion in MCF-7 cells. Similarly, in osteoblasts, inhibition of AQP1 *via* miR-495 overexpression activated p38 MAPK signaling and promoted proliferation and differentiation of osteoblasts in mice with tibial fracture (105). Because bone is the most common site for breast cancer metastasis, targeting AQP1 with miR-495 could be therapeutically beneficial and reduce bone destruction in breast cancer patients.

### Aquaporin-3

AQP3 also plays a key role in cancer progression in cells and tumors derived from several cancer subtypes. AQP3 was required for cell migration and invasion but not proliferation in T47D and MCF-7 cells (92). Estrogen receptor positive (ER+) breast cancer tissues with high AQP3 expression were associated with poorer cell differentiation and increased lymph node metastasis (92). Additionally, estrogen could promote AQP3 upregulation by activating an estrogen response element (ERE) in the promoter region of AQP3. AQP3 overexpression increased cell migration and invasion by regulating EMT-related factors gene expression and reorganization of actin cytoskeleton.

In triple negative breast cancer cells MDA-MB-231, AQP3 inhibition by shRNA decreased cellular water and glycerol permeability, cell proliferation, migration, and invasion (93). Additional studies in MDA-MB-231 and DU4475 breast cancer cells found AQP3 to be critical for cell migration through cell signaling. For example, the hydrogen peroxide transport function of AQP3 was essential for CXCL12/CXCR4-dependent cell migration through PTEN, protein tyrosine phosphatase 1B (PTP1B), and Akt signaling (94). Overexpression of AQP3 increased H_2_O_2_ uptake and cell migration in response to CXCL12 stimulation. Also, *via* an alternative mechanism, AQP3 was required for fibroblast growth factor-2 (FGF-2)-induced cell migration *via* the FGFR-PI3K and FGFR-ERK signal transduction pathways in MDA-MB-231 and Bcap-37 cells (**Figure 2**) (95).

In HER2+ early breast cancer, AQP3 overexpression was associated with poor prognosis and poor recurrence-free survival (96). AQP3 overexpression in TNBC was prognostic of poor five-year disease-free survival and overall survival as well as tumor size, lymph node status, and metastasis (82).

### Aquaporin-4

Downregulation of AQP4 by siRNA in T47D and MCF-7 cells decreased cell proliferation, migration, and invasion (97). In addition, knockdown of AQP4 increased E-cadherin transcript and protein expression, indicating a regulatory role for AQP4. ERK signaling was increased by AQP4 knockdown, where levels of phosphorylated ERK increased significantly.

### Aquaporin-5

AQP5 has prognostic value in breast cancer patient samples. In ER+/progesterone receptor (PR)+ early breast cancer, AQP5 overexpression is a potential prognostic marker of patient survival (98). In TNBC, AQP5 overexpression correlated with tumor size, nodal status, local relapse/distant metastasis, and elevated Ki-67 expression (82). AQP5 expression also significantly correlated with downstream signaling molecules Rac1 and Ras, a Rho-family member GTPase, and other contributors to cancer migration (99). In invasive ductal carcinoma (IDC) patient tissues, high AQP5 expression did not correlate with Ras but positively correlated with Rac1, which makes Rac1 a candidate downstream target of AQP5 in breast cancer (99).

In cell culture, AQP5 knockdown by shRNA decreased cell proliferation and migration in response to sorbitol-induced hyperosmotic stress in MCF-7 cells (100). Similarly, AQP5-targeting miRNAs significantly reduced AQP5 expression and migration of MDA-MB-231 cells (101).

### Aquaporin-7

We identified AQP7 as a negative prognostic marker of overall survival and metastasis in breast cancer patients (19). Functional studies of Aqp7 in cultured 4T1 cells highlighted roles for Aqp7 in regulating proliferation, contact inhibition, and adhesion. Aqp7 knockdown decreased proliferation, increased contact inhibition, and increased adhesion. Aqp7 knockdown also significantly reduced primary tumor burden and metastasis. Metabolomics of Aqp7 knockdown cells and tumors revealed significantly altered lipid levels, with an accumulation of lipids in tumors with Aqp7 knockdown. Aqp7 knockdown also caused a redox imbalance, where cells were sensitized to oxidizing environments. In urea/arginine metabolism, Aqp7 knockdown reduced nitric oxide production and iNOS expression, while increasing arginine accumulation and arginase 1 expression. These data support AQP7 as a critical regulator of metabolic and signaling responses to environmental cellular stresses in breast cancer, making AQP7 an attractive therapeutic target (19).

## The role of aquaporins in the tumor microenvironment

**Figure 3:**
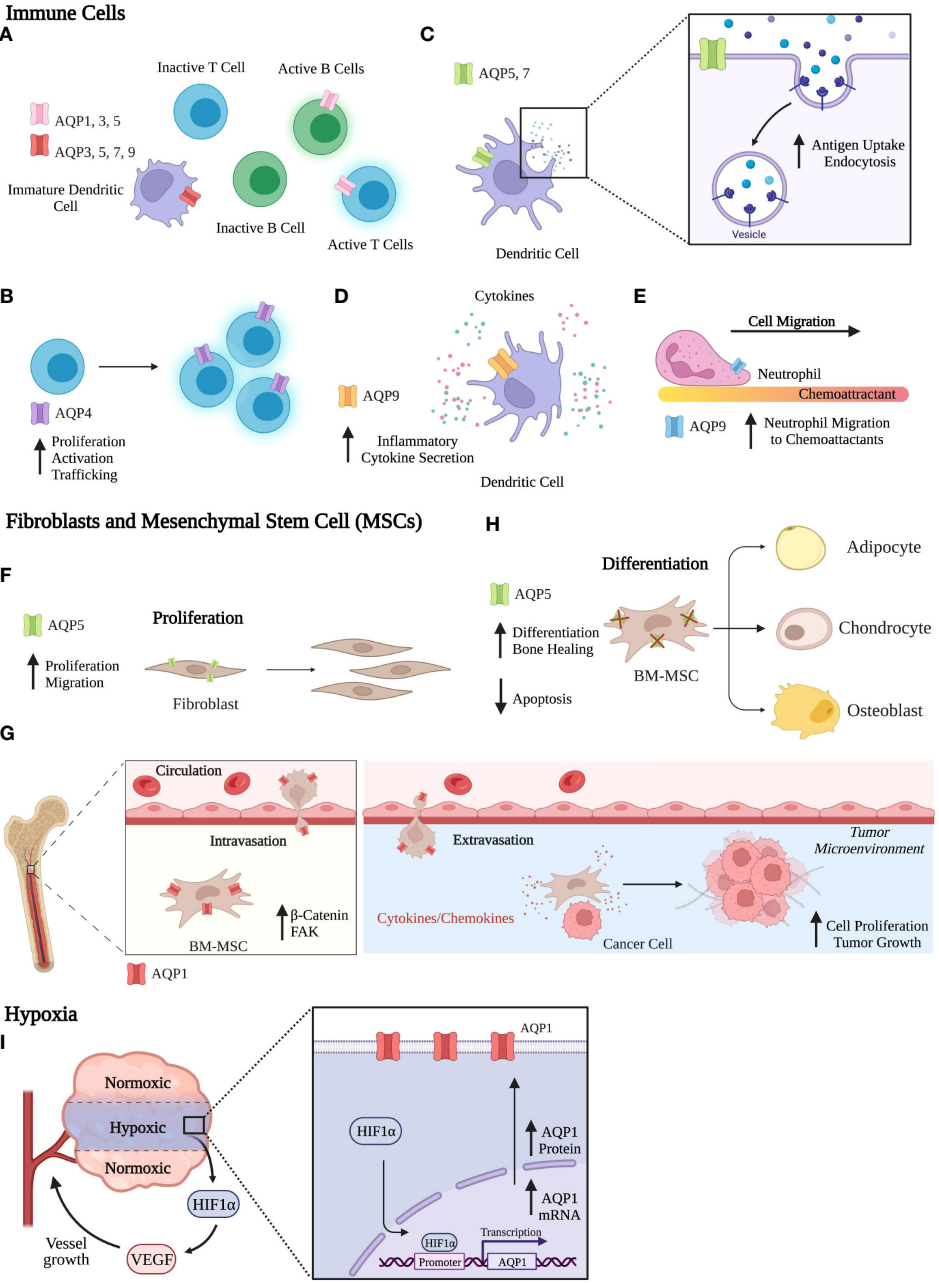
Functional Roles of Aquaporins. **(A-E)** *Immune Cells*. **(A)** AQP expression across various immune cells. **(B)** AQP4 is involved in T cell activation and proliferation. **(C)** In dendritic cells, AQP5 and AQP7 contribute to antigen uptake and endocytosis. **(D)** In dendritic cells, AQP9 expression aids in secretion of inflammatory cytokines. **(E)** AQP9 increases neutrophil migration to chemoattractants. **(F-H)** Fibroblasts and MSCs. **(F)** AQP5 expression in fibroblasts increases proliferation and migration. **(G)** AQP1 expression in BM-MSCs aids in migration, where BM-MSCs can interact with the TME to promote breast cancer progression. **(H)** AQP5 knockout in BM-MSCs increases differentiation and bone healing and reduces apoptosis rates. **(I)** *Hypoxia.* AQP1 mRNA and protein expression increase in response to hypoxia. Created with BioRender.com.

Breast cancer cells are highly dependent on interactions between different components of the microenvironment for their survival, proliferation, and progression as cancer. The tumor microenvironment (TME) has a profound effect on breast cancer development, progression, and response to therapeutics (106–108). Apart from cancer cells, the TME includes cells of the immune system, fibroblasts, stromal cells, vasculature, and adipocytes. Microenvironmental factors within the tumor, such as nutrient availability, cellular interactions, and the extracellular matrix, can influence the behavior of tumor cells (106, 109). The crosstalk between tumor cells and the TME is coordinated with reprogrammed cellular signaling and epigenetics to promote breast cancer development, heterogeneity, metastasis, and ultimately drug resistance. Several reviews have focused on the role of AQPs in adipocytes and roles in migration, adhesion, epithelial-mesenchymal transition (EMT), and angiogenesis (17, 20, 61, 74, 89). However, no *in vivo* studies have compared the contributions of stromal-derived to epithelial-derived AQPs to breast cancer. Below we focus on the role of AQPs in other components of the TME, including the immune cells, fibroblasts, stromal cells, and hypoxia (**Figure 3**).

### Immune cells

The immune landscape has a considerable impact on breast cancer progression and metastasis, where immune cells can kill neoplastic cells, prevent tumor progression, and shape tumor immunogenicity by contributing to the tumor microenvironment. AQPs are involved in several processes related to immunity and inflammation, including priming and inflammasome activation, transendothelial migration, and phagocytosis.

AQPs are expressed in both myeloid and lymphoid cell populations. In lymphoid cells, AQP1, AQP3, and AQP5 are expressed in human activated B and T lymphocytes, while no AQPs are expressed in inactivated B and T lymphocytes by RT-qPCR analysis (**Figure 3A**) (110). Expression of AQP2 and AQP8 were not observed in lymphoid cells. AQP4 is expressed by both naïve and memory CD4 and CD8 T cells (111). Inhibition of AQP4 using AER-270/271, small molecule inhibitors, directly affects T lymphocytes by reducing T cell activation, proliferation, and trafficking (**Figure 3B**) (112). In breast cancer, AQP9 expression is positively correlated with immune infiltrates, such as B cells, CD4+ and CD8+T cells, neutrophils, macrophages, and dendritic cells (DCs) (104).

In myeloid cells, AQP3, AQP5, AQP7, and AQP9 are expressed in immature DCs, whereas AQP1 and AQP2 were absent (**Figure 3A**) (110, 113). Upon maturation of DCs, AQP3 and AQP7 expressions are downregulated, whereas AQP9 is expressed throughout DC development from monocytes to mature DCs (113). DCs treated with pCMBS, a mercuric drug that inhibits AQP channel activity, had reduced uptake and accumulation of macrosolutes taken up by fluid-phase endocytosis but not by receptor mediated endocytosis. Additionally, pCMBS-treated DCs exhibited increased swelling. These results indicate that AQPs are essential in volume regulation where DCs avoid swelling by eliminating excess fluid taken up by micropinocytosis. Ablation of AQP5 and AQP7 expression decreased both antigen uptake and endocytosis in DCs (**Figure 3C**) (114–116). Using AQP7 knockout in mice, AQP7 was required for chemokine-dependent migration, antigen uptake, and processing (115). In murine bone marrow-derived DCs, AQP9 was the most highly expressed AQP protein after lipopolysaccharide exposure, and inhibition of AQP9 decreased inflammatory cytokine secretion (**Figure 3D**) (117). In breast cancer patient datasets, AQP9 expression significantly correlated with specific markers, including eCD86 and CD115 (monocytes); CD68, IL10, and CCL-2 (TAMs); IRF5 and PTGS2 (M1 macrophages); and CD163, MS4A4A, and VSIG4 (M2 macrophages) (104).

AQPs contribute to the migration of multiple immune cell populations. Interestingly, AQP3, AQP5, and AQP9 are critical in the immune system due to their role in immune cell migration, where AQP5 and AQP9 regulate neutrophil cell migration and impact sepsis survival (116). AQP9 localization on the cell edge enables cells to move toward chemoattractants by facilitating motility, lamellipodium extension and stabilization, and cell volume changes (**Figure 3E**) (118). In T cells, AQP3-mediated H_2_O_2_ transport was essential for T cell migration toward chemokines (119).

### Fibroblasts and mesenchymal stem cells

In non-cancerous fibroblast cell lines, AQP5 overexpression increased proliferation and cell movement in an invasion assay (**Figure 3F**) (120). Additionally, overexpression of wildtype AQP5 in cancer cells and fibroblasts activated ERK 1/2 (102). However, a mutation in the D loop (S156A) abolished ERK 1/2 activation and downstream targets Rac1 and RhoA proteins, which regulate cell motility (102, 121). AQP5-S156A overexpression increased cell proliferation compared to WT AQP5. In fibroblasts, AQP5 overexpression induced tumor formation in nude mice, while AQP5-S156A mutation abolished tumor formation (120). Given the influence of phosphorylation on the PKA consensus site of AQP5 and its effect on cell proliferation, we can associate AQP5 expression with signaling transduction pathways.

Mesenchymal stem cells (MSCs) play a critical role in tumor progression. In both human and mouse tumor models, MSCs can be recruited to the TME, where they differentiate into CAFs or other stromal cell types that promote growth and angiogenesis in breast cancer (122). MSCs also secrete a plethora of pro-stemness cytokines and growth factors that can promote tumor progression and metastasis (123).

The role of MSCs on breast cancer cells can be recapitulated in cell culture. Proliferation increased in 4T1 mouse mammary tumor cells co-cultured with either bone marrow mesenchymal stem cells (BM-MSCs) or BM-MSC conditioned media (122). Subcutaneous injection of 4T1 cells co-injected with BM-MSCs had increased tumor growth compared to 4T1 cell injection alone in nude mice. Overexpression of AQP1 enhanced the migration of BM-MSCs by upregulating the protein expression of β-catenin and focal adhesion kinase (FAK), regulators of cell migration (**Figure 3G**) (124). Using conditioned media from BM-MSCs, AQP1 protein expression increased in osteosarcomas and hepatocellular carcinoma after 24hrs of treatment (125). Wound healing and cell invasiveness was increased after treatment with BM-MSC conditioned media compared to control. The addition of an AQP1 inhibitor, tetraethylammonium chloride (TEA), hampered these effects, confirming AQP1 involvement. AQP5 was expressed in BM-MSCs on the plasma membrane to mediate water permeability and was required for apoptosis of differentiating BM-MSCs (126). Characterization of BM-MSC differentiation capacity showed that AQP5 knockout in mice increased adipogenic, osteogenic, and chondrogenic differentiation compared to WT BM-MSCs (**Figure 3H**). In a drill-hole injury model, bone healing was accelerated in AQP5 knockout mice compared to WT mice.

### Hypoxia

Hypoxic regions of the tumor microenvironment have restricted access to nutrients and oxygen due to aberrant vascularization and poor blood supply. In response, cells from the primary tumor can produce hypoxia-inducible factors (HIFs), in particular HIF-1α which reprograms tumor cells and signaling pathways and regulates oncogenesis, angiogenesis, and metastasis (127). Using a panel of 155 female breast cancer tissues, AQP1 and HIF-1α expression were stained by immunohistochemistry and analyzed (90). Tissues expressing HIF-1α had higher AQP1 expression compared to HIF-1α negative tissues, suggesting that AQP1 and HIF-1α might functionally interact (90). In fact, AQP1 mRNA and protein expression were induced in hypoxia by HIF-1α in mouse brain and lungs and cultured cells (128). In cultured human retinal vascular endothelial cells, the AQP1 gene promoter contains a HIF-1α binding site that regulates AQP1 expression in response to hypoxia (**Figure 3I**) (129).

## Aquaporins as predictive biomarkers and targeted therapeutics

In the past five years, AQPs have emerged as potential predictors of response to cancer therapy and as targets for increasing sensitivity to treatment. Both expression levels and localization of the AQPs contribute to AQP function and to downstream signaling to regulate treatment response. However, cell-based assays that evaluate the requirements for AQPs in developing resistance to standard breast cancer treatment have not been evaluated. Also, inhibitors of AQPs have not yet been tested for their efficacy in treating breast cancer. We will describe evidence that provides a rationale for further research into how AQPs contribute to therapeutic resistance.

### AQP1

AQPs contribute significantly to chemotherapy response. In IDC patients with increased expression of AQP1, they had better clinical outcomes to anthracycline treatment compared to those with low AQP1 expression (91). Also, miR-320a-3p could inhibit AQP1 expression and attenuate chemosensitivity to epirubicin. Investigating the mechanism regulating chemosensitivity uncovered that cytoplasmic AQP1 and glycogen synthase kinase-3β (GSK3β) interact *via* 12 armadillo repeats of β-catenin, leading to β-catenin accumulation and its interaction with Topo IIα and enhancing its activity, which increased the sensitivity to anthracycline treatment (91). This association with chemotherapy sensitivity *via* AQP1 occurred not only in breast cancer but also in human bladder cancer cells, where AQP1 inhibition enhanced mitomycin C sensitivity, and also in colorectal and ovarian cancers (130–132).

### AQP5

AQP5 affects the chemosensitivity of colorectal cancer cells (CRC), where AQP5 expression is upregulated in CRCs and 5-FU-resistant cells. The miR-185-3p not only targets and regulates AQP5 but also enhances the sensitivity of CRCs to 5-FU *via* the Wnt-β-catenin signaling pathway and EMT progress (133). While AQP5 silencing inhibited Wnt-β-catenin signaling, overexpression of the degradation-resistant mutant of β-catenin (S33Y) reversed apoptosis induced by AQP5 silencing (134). Future studies will need to determine if similar mechanisms of chemosensitivity occur in breast cancer cells. In adriamycin (ADR)-resistant breast cancer cell line MCF-7 (MCF-7/ADR), AQP5 inhibition inhibited proliferation and induced apoptosis (103). AQP5 inhibition also reversed ADR resistance of breast cancer and reduced the IC50 of ADR in MCF-7/ADR cells.

### AQPs as prognostic and predictive markers in patients

**Figure 4:**
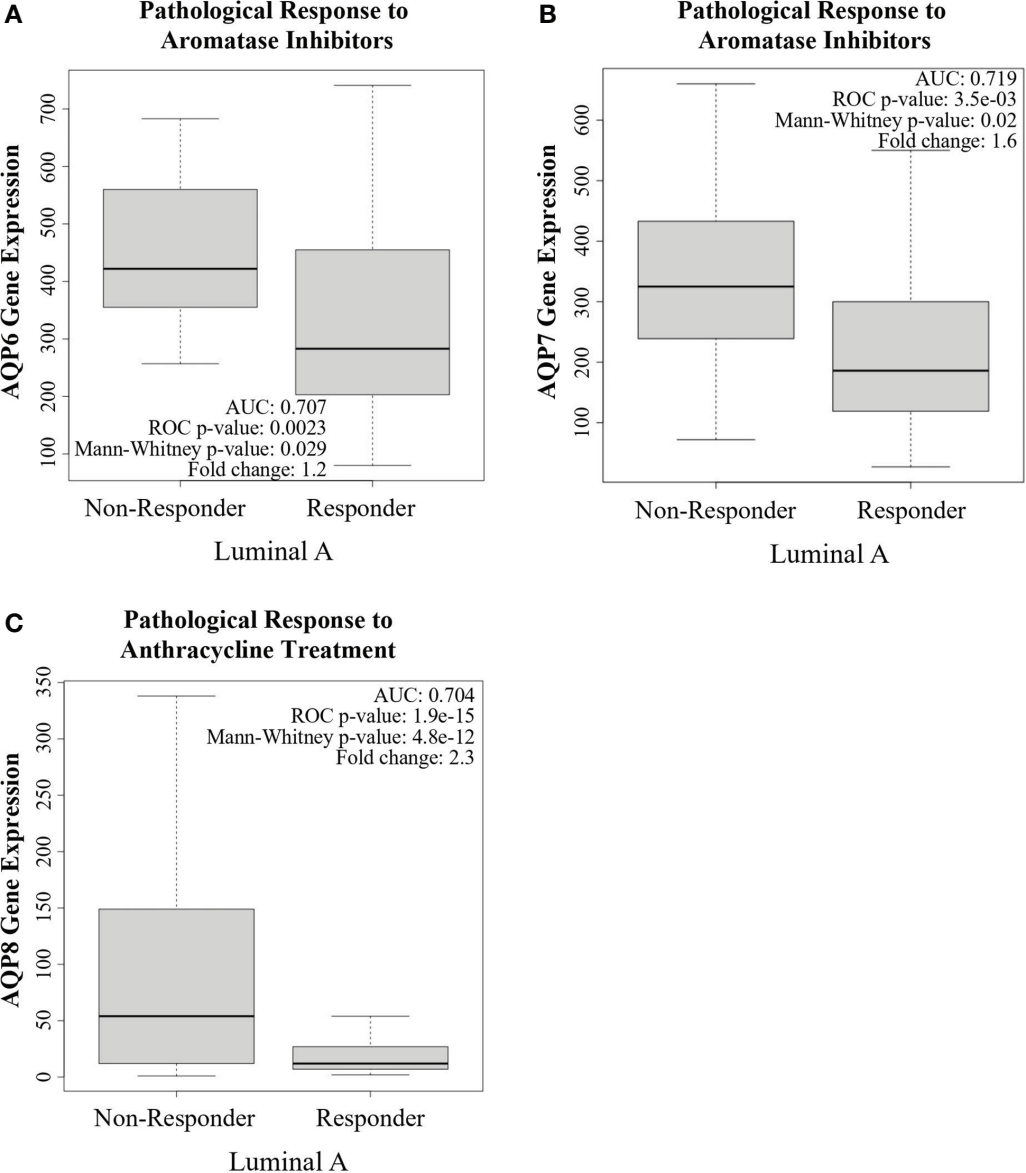
Aquaporins as a potential predictive biomarker. ROC plotter analysis of AQP6, AQP7, and AQP8 gene expression in treatment responders and non-responders, according to pathological complete response in breast cancer patients with Luminal A subtype tumors and the indicated treatments. **(A)** AQP6 expression was lower in responders to aromatase inhibitors than in non-responders. **(B)** AQP7 expression was lower in responders to aromatase inhibitors than in non-responders. **(C)** AQP8 expression was lower in responders to anthracycline treatment than in non-responders.

Given the recent evidence supporting a role for AQPs as potential predictive markers, we evaluated the predictive ability of AQPs in breast cancer using ROC plotter, an online transcriptome-level validation tool for predictive biomarkers (135). We evaluated 13 AQPs by ROC analysis, which links gene expression and pathological complete response to therapy. This analysis identified AQPs as predictive biomarkers (**Table 2**). The most stringent analysis identified AQP6, AQP7, and AQP8 as potential quality cancer biomarkers with an area under the curve (AUC) above 0.7. Additionally, four AQPs (AQP2, AQP4, AQP10, and AQP12) were identified as biomarkers with potential clinical utility (AUC between 0.6-0.7), according to pathological complete response to either endocrine therapy, HER2-targeting therapy, or chemotherapy (**Table 2**). Of these AQPs, reduced expressions of AQP6 or AQP7 were associated with responders to aromatase inhibitors for Luminal A subtype breast cancer, whereas reduced AQP8 expression was associated with responders to anthracycline treatment for Luminal A subtype breast cancer (**Figures 4A–C**).

**Table 2:** ROC Analysis of Pathological Complete Response of AQPs for Breast Cancer. ROC Plotter links gene expression and response to therapy using transcriptome-level data of breast cancer patients.

Gene	AQP Expression for Responders	Treatment	Subtype	AUC	ROC p value	Mann Whitney t test	Fold change	Dataset
AQP2	High	Any anti-HER2	All	0.605	0.0036	0.0083	1.3	206672_at
AQP2	High	Any anti-HER2	HER2+	0.611	0.0071	0.016	1.4	206672_at
AQP4	High	Any anti-HER2	All	0.633	0.00022	0.00078	1.2	210066_s_at
AQP6	Low	Aromatase Inhibitor	Luminal A	0.707	0.0023	0.029	1.2	216219_at
AQP6	High	Any anti-HER2	All	0.63	0.00028	0.001	1.2	216219_at
AQP6	High	Any anti-HER2	HER2+ER-	0.686	0.0012	0.0054	1.6	216219_at
AQP6	High	Trastuzumab	HER2+ER-	0.673	0.0056	0.018	1.5	216219_at
AQP6	Low	Any Endocrine	All	0.687	0.0038	0.019	1.2	216219_at
AQP6	High	Any anti-HER2	All	0.605	0.0031	0.0079	1.1	208435_s_at
AQP6	Low	Any Chemotherapy	All	0.603	1.00E-11	1.60E-11	1.3	208435_s_at
AQP7	Low	Anthracycline	TNBC	0.624	8.50E-05	0.00028	1.2	206955_at
AQP7	High	Trastuzumab	HER2+	0.605	1.70E-02	0.037	1.3	206955_at
AQP7	Low	Aromatase Inhibitor	Luminal A	0.719	3.50E-03	0.02	1.6	206955_at
AQP7	Low	Any Endocrine	All	0.677	0.0097	0.027	1.6	206955_at
AQP7	Low	Any Endocrine	Luminal A	0.719	3.50E-03	0.02	1.6	206955_at
AQP8	Low	Anthracycline	Luminal A	0.704	1.90E-15	4.90E-12	2.3	206784_at
AQP8	Low	Taxane	Luminal A	0.662	0.00000039	0.0000053	1.8	206784_at
AQP8	High	Taxane	HER2+ER-	0.63	0.0026	0.0076	1.4	206784_at
AQP10	High	Any Chemotherapy	Luminal B	0.634	0.013	0.03	1.6	1555338_s_at*
AQP10	High	Anthracycline	HER2+ER-	0.624	0.015	0.044	1.3	1555338_s_at*
AQP10	High	Any anti-HER2	HER2+	0.632	0.009	0.024	1.6	1555338_s_at*
AQP10	High	Any anti-HER2	All	0.608	0.0097	0.026	1.2	1555338_s_at*
AQP12B	High	Any Chemotherapy	All	0.614	0.000072	0.00016	1.5	1559575_a_at*
AQP12B/AQP12A	High	Any Chemotherapy	All	0.617	0.000061	0.00011	1.3	1554344_s_at*
AQP12B/AQP12A	High	Any anti-HER2	All	0.605	0.013	0.031	1.3	1554344_s_at*

Area under the curve (AUC) indicates prognostic power of gene. 0.6-0.7, cancer biomarker with potential clinical utility, 0.7-0.8, top quality cancer biomarker, and 0.8+, blockbuster biomarker. AQPs with an AUC > 0.6 and P<0.05, Mann Whitney t test.

### AQP inhibitors

These correlations between AQP expression and their roles in breast cancer progression and treatment response are strong evidence supporting a role for AQPs in response to treatment and AQP targeting agents as potential therapeutics. As a result, small molecules targeting AQPs have been investigated for their efficacy and clinical potential in treating several diseases, including cancer. While the field of AQP-based therapeutics has grown in number, the identification of AQP inhibitors for therapeutic applications has been extremely challenging. **Table 3** includes a list of AQP inhibitors currently available.

**Table 3:** Aquaporin Inhibitors. List of available AQP inhibitors and the AQPs they inhibit.

AQP	Inhibitors in human	Inhibitors in mouse	Inhibitors in rat
AQP1	Tetraethylammonium (136), HgCl_2_ (25), AqB007, AqB011 (137), AqB013 (138), acetazolamide, test compounds 1-3 (139), HAuCl_4_, AgNO_3_ (140), *p*-chloromercuribenzoate, DMSO (141), AqF026 (142), Bacopasides (143), furosemide (144)	HgCl_2_ (145), HAuCl_4_, AgNO_3_ (140)	HgCl_2_, p-hydroxymercuribenzoic sulfonic acid (146), acetazolamide (147)
AQP2		Phenylbenzamides (148)	
AQP3	HgCl_2_ (149), Phloretin (150), Auphen (151), CuSO_4_ (152), NiCl_2_ (153), DFP00173, Z433927330 (154), polyoxotungstate-Agold (III) complexes (155), gold (III)-Bipyridyl compounds (156)	HgCl_2_ (157), DFP00173 (154)	Phloretin, *p*-chloromercuriphenylsulfonate (158), HgCl_2_, CuSO_4_ (159)
AQP4	AER-270 (160), 2-(nicotinamido)-1,3,4-thiadiazole, sumatriptan, rizatriptan (161), acetazolamide, 6-Ethoxybenzothiazole-2-sulfonamide (162), 1,3-Oxazoles, Oxazoles with condensed carbocyclic rings, 1,3-Thiazoles, Thiazoles, Thiazoles with condensed carbocyclic rings (163), Topiramate, Zonisamide, Phenytoin, Oxcarbazepine, Lamotrigine, Valproic acid, Carbamazepine-10,11-epoxide (162)	Phenylbenzamides (148)	HgCl_2_ (164), AqB013, bumetanide (138), acetazolamide (165)
AQP5	CuSO_4_ (166), HgCl_2_ (167)	HgCl_2_ (168)	HgCl_2_ (169)
AQP7	Auphen (170), monoacetin, monobutyrin, diacetin (171)	Auphen (170) Z433927330 (154)	
AQP8	HgCl_2_ (172)	HgCl_2_ (172)	HgCl_2_ (172, 173), CuSO_4_ (174)
AQP9	Phloretin, HgCl_2_ (175, 176), nigericin, carbonyl cyanide 4-trifluoromethoxyphenylhydrazone (177) compounds ID4-6 (178)	Phloretin (179), HTS13286 (180)	Phloretin (181)
AQP10	HgCl_2_ (182), organometallic gold complexes (183)		
AQP11		HgCl_2_ (184)	

The development of candidate AQP modulators for clinical use is still lacking in part because many AQP modulators are not AQP specific or because of compensation by other AQPs. Several AQP inhibitors, like DF00173, Z433927330, and RF03176, have been identified with potential selectivity for AQP3, AQP7, and AQP9 respectively (185–187). *In vivo* studies of AQP1 inhibitor, bacopaside ll, and AQP4 inhibitors, AER-270 and TGN-020, show promise for treating cardiomyopathies and brain and central nervous system (CNS) edema (188). A pro-drug AER-271 is currently in phase l clinical trials with no update on data.

While some AQP inhibitors were identified by screening a library of compounds, multiple small molecule inhibitors of AQPs have been identified using computational chemistry methods. The use of molecular docking and dynamic simulations have been used to screen libraries and to gain mechanistic insight into channel inhibition by these compounds. The gold (III) compound Auphen is an aquaporin inhibitor of AQP3, AQP7, and AQP9 and was efficacious in treating hepatocellular carcinoma by regulating AQP3 expression (189). Docking studies identified the thioether groups of methionine residues as binding sites of Auphen to AQP7 (170). Some of the identified AQP inhibitors appear to bind to the extracellular entrance close to ar/R residues or near the intracellular pore entrance (139, 170, 178). Computational methods have provided remarkable insight into AQP drug discovery with hopes that analogues of these identified inhibitors will improve their efficacy as therapeutics. However, the pharmacokinetic and pharmacodynamic properties of many of these AQP inhibitors, identified either by computational or experimental screens, have yet to be studied and tested. In addition, future studies will be needed to consider compensation by other AQPs in evaluating their efficacy.

While inhibition of various AQPs in breast cancer reduces pro-tumorigenic phenotypes, including tumor growth and metastasis, inhibitors like the ones mentioned above have yet to be characterized for their efficacy in treating breast cancer. No studies have characterized the effect of AQP inhibitors as a component of a combination treatment to demonstrate a causal relationship between AQPs and treatment, or whether combination treatment can overcome breast cancer therapeutic resistance, indicating an area in the field that needs further research. In addition, add back experiments with AQPs that restore cancer progression will further validate the specific contributions of AQPs to the process.

Several recent reviews summarized potential aquaporin-modulating small molecules and biologics and the challenges and opportunities in developing AQP-based therapeutics (83, 185–188). These compounds represent a starting point in which further investigation and development of these inhibitors are needed to create suitable compounds for pharmacological evaluation in cell culture and *in vivo*. A better understanding of AQP structure and function also will shed light onto how these transport proteins promote cell growth and activity and how their dysregulation can lead to disease and response to cancer treatments.

## Conclusion and future directions

In conclusion, research over the last decade has created a growing interest in AQPs due to their ability to transport not only water but also other small molecules, like glycerol and hydrogen peroxide. Control of glycerol transport by aquaglyceroporins plays a crucial role in the dysregulation of these glycerol channels and their association with metabolic diseases, such as obesity, insulin resistance, and cancer. AQPs play important biological functions in breast cancer, contributing to critical cellular processes such as cell proliferation, migration, and tumor growth.

AQPs are proposed as prognostic indicators with the potential of being useful predictive markers to help guide treatment strategies with increased efficacy. We are only beginning to understand the importance and role of AQPs in breast cancer and other TME factors. However, further research and mechanistic analysis of how AQPs contribute to these processes still need to be elucidated to discover new markers for prognostic and therapeutic use.

Even though AQPs have significant roles that contribute to various human diseases, very little progress has been made in identifying AQP modulators or biomarker assays for clinical use in any of these diseases, including cancer. The literature has a dearth of characterization of the inhibitors. Metabolite stability assays and pharmacokinetics will be important in moving AQPs and their inhibitors in a clinically relevant direction. To rationally design AQP modulators, particularly ones that are isoform-selective, we need a better understanding of AQP structure and function. Mutants that were discussed earlier in this review that are generated for structure-function analysis also should be used to reveal AQP structural features that impact breast cancer progression, metastasis, and response to treatments. Successful completion of this study using both cell culture and *in vivo* experimental approaches will provide insight into the importance of these structural features.

Given the contributions of AQP function in stromal cells like adipocytes and immune cells, it is surprising that no studies have distinguished the roles between stromal-derived and cancer cell-derived AQPs or evaluated their role in mammary gland development. Indeed, the requirements for stromal-derived AQPs *in vivo* have not been analyzed in the context of mammary cancer. Both genetic inhibition of AQPs and their systemic therapeutic inhibition using pan-AQP, and AQP-specific inhibitors should be tested for their efficacy on mammary cancer progression, metastasis, and ability to overcome resistance to standard therapies. Evaluating the *in vivo* genetic requirements for AQPs will be particularly valuable to determine if there is a clinical rationale for using a general aquaporin inhibitor or epithelial-targeted (which will require delivery strategies to be evaluated) inhibition of AQPs to target metastatic breast cancer.

Certainly, if any of the AQP inhibitors have efficacy against breast cancer tumor burden in preclinical animal models, then their treatment should be compared as single agents and in combination with standard of care chemotherapy and other targeted treatments for efficacy in to overcoming metastatic disease or therapy resistance. In addition, future directions of this research also will likely include additional chemical optimization of the AQP inhibitors to have increased solubility, selectivity, and potency against specific AQPs.

## Author contributions

Authors indicated in parenthesis made substantial contributions to the following tasks: writing (VC, DF, CA, ZW, KC, and LL); contribution of figures and tables (VC, DF, CA, and LL); revision of paper (VC, CA, and LL). All authors contributed to the article and approved the submitted version.

## Funding

VC is a fellow of the Chemistry-Biochemistry-Biology Interface (CBBI) Program at the University of Notre Dame, supported by training grant T32GM075762 from the National Institute of General Medical Sciences. VC currently is supported by the Dean’s Fellowship from the University of Notre Dame. LL is supported by the National Institutes of Health and the National Cancer Institute (CA252878, CA237607), the DOD BCRP Breakthrough Award, Level 2 (W81XWH2110432), and the Catherine Peachey Fund with the Heroes Foundation.

## Conflict of interest

The authors declare that the research was conducted in the absence of any commercial or financial relationships that could be construed as a potential conflict of interest.

## Publisher’s note

All claims expressed in this article are solely those of the authors and do not necessarily represent those of their affiliated organizations, or those of the publisher, the editors and the reviewers. Any product that may be evaluated in this article, or claim that may be made by its manufacturer, is not guaranteed or endorsed by the publisher.
